# Evaluation of blood-brain barrier permeability in tryptophan hydroxylase 2-knockout mice

**DOI:** 10.3892/etm.2014.1938

**Published:** 2014-08-28

**Authors:** CHAO-JIN XU, PENG-ZHANG, TING-LI DAI, XUE-YUAN NIU, JUN-LING WANG, MING-SHUN JIN, MIN-LIAO

**Affiliations:** 1 Department of Histology and Embryology, Institute of Neuroscience, Wenzhou Medical University, Wenzhou, Zhejiang 325035, P.R. China; 2 Department of Anatomy, Wenzhou Medical University, Wenzhou, Zhejiang 325035, P.R. China; 3 School of Laboratory Medicine and Life Science, Wenzhou Medical University, Wenzhou, Zhejiang 325035, P.R. China

**Keywords:** blood-brain barrier, tryptophan hydroxylase 2, 5-hydroxytryptamine

## Abstract

The blood-brain barrier (BBB) is critical to the health of the central nervous system (CNS). The possibility that 5-hydroxytryptamine (5-HT) participates in the alteration of the BBB has been previously demonstrated. Tryptophan hydroxylase 2 (TPH2) is a unique genetic enzyme isoform that catalyzes the rate-limiting step in the biosynthesis of 5-HT in the CNS; however, its role in the permeability changes of the BBB remains unclear. In the present study, TPH2-knockout mice were utilized in the assessment of BBB disruption, as measured by the Evans Blue (EB) extravasation or fluorescein isothiocyanate-albumin leakage assay in the brain. EB was not found to be retained in the brain in the TPH2-knockout mice or the wild-type controls. The results of the study demonstrate that TPH2 knockout has no effect on BBB permeability, indicating that TPH2 and the 5-HT system in the CNS are not sufficient to influence the BBB leakage.

## Introduction

The blood-brain barrier (BBB) plays an important role in maintaining a stable environment in normal brain and spinal cord function. Changes in BBB permeability have been described in several pathological conditions, including poisoning, immune insults and irradiation, as well as in selected neurological disorders, such as stroke, traumatic brain injury and spinal cord injury (1), where the parenchyma of the brain or spinal cord is severely damaged (2). Additionally, there have been studies demonstrating that disruption of the BBB can occur in certain depressive disorders (3,4), including 5-hydroxytryptamine (5-HT)-related diseases. For example, abnormal levels of 5-HT have been demonstrated to result in neuronal malfunction, and a genetics study showed that mice with insufficient 5-HT exhibited anxiety and aggressive behavior (5). A number of studies have also reported that antibodies against 5-HT (6), inhibitors of 5-HT synthesis (7) and 5-HT-modulating compounds (8) can influence the permeability of the BBB. 5-HT is synthesized from L-tryptophan in two steps which are catalyzed by tryptophan hydroxylase (TPH). Thus, TPH is able to regulate 5-HT in the peripheral tissues and central nervous system. The genes encoding Tph1 and Tph2 are located on chromosomes 7B5 and 10D1 in the mouse (9). TPH1 is mainly expressed and synthesized in the periphery (10), but TPH2 is preferentially synthesized in the brain. However, the effect of tryptophan hydroxylase 2 (TPH2), a rate-limiting enzyme of 5-HT biosynthesis, on the integrity of the BBB remains unclear. Therefore, the present experiment investigated the effect of TPH2 on BBB disruption. BBB permeability was evaluated by Evans blue (EB) staining in TPH2-knockout mice.

## Materials and methods

### Materials

EB (E2129-1G) was purchased from Sigma-Aldrich (St. Louis, MO, USA). PL2000 DNA marker (D501A; 2,000 bp) was purchased from Takara Biotechnology, Co., Ltd. (Dalian, China). Anti-TPH2 antibody (PA1-778) was purchased from Thermo Fisher Scientific, Inc. (Waltham, MA, USA). Fluorescein isothiocyanate (FITC)-albumin (A9771) was purchased from Sigma-Aldrich. Wild-type (C57BL/6) mice were crossed with heterozygous TPH2-flox mice and their offspring generated homozygous TPH2-knockout mice.

### Animals and treatment

All animal protocols used in this study were approved by the Animal Committee of Tongji University School of Medicine (TJmed-010–10; Shanghai, China) (11). Adult (12 weeks old, weighing ~25 g) TPH2-knockout (n=6) and wild-type (n=6) mice were used for this study. For immunocytochemistry (n=3 from each group) (12), mice were anesthetized with pentobarbital [50 mg/kg, intraperitoneal (i.p.)] prior to undergoing transcardial perfusion with 4% paraformaldehyde. The brain was removed and placed in a 10% sucrose solution overnight. The following day, the brain was placed in a 20% sucrose solution for 2 h and then transferred into a 30% sucrose solution. The brain was subsequently sectioned on a microtome at a thickness of 40 μm. The sections through the brainstem were collected in a cell culture plate containing cryoprotectant [30% glycerol, 30% ethylene glycol and 40 μm phosphate-buffered saline (PBS)] (13). Serial sections were collected and placed individually into each of the six wells. This sectioning protocol resulted in six series of sections in total (~40 sections/series) through the brainstem that were 240 μm apart (40 μm × 6). Two wells from each animal were assayed for TPH2 expression.

### Immunocytochemistry

Brain sections (40-mm) were incubated with primary antibody (anti-TPH2; 1:1,000) (14) at 4°C overnight. Subsequent to washing in PBS, the sections were incubated with rhodamine-conjugated affinity pure goat anti-rabbit immunoglobulin G (IgG) (Heavy and Light chain; 1:100; Jackson ImmunoResearch Laboratories, Inc., West Grove, PA, USA) secondary antibody for 2 h at room temperature and then washed in PBS. No immunostaining signals were observed when the primary antibody was omitted or replaced with normal IgG. Stained sections were observed and scanned under a fluorescence microscope (Olympus BX53; Olympus, Tokyo, Japan).

### EB extravasation

The BBB permeability was measured using EB (14). In brief, TPH2-knockout mice and their age-matched controls were weighed and injected (i.p.) with 50 μg/g EB dye in PBS. Twelve hours after injection, the mice were anesthetized and perfused with PBS for 5 min. Following perfusion, the brains were dissected and the olfactory bulbs and cerebella were removed. For the inspection of EB extravasation, the brains were placed in PBS containing 30% sucrose overnight, and 40-μm coronal sections were then cut on a cryostat and mounted onto gelatin-coated glass slides. All mice used in this study exhibited high levels of EB dye in the liver.

### FITC-albumin leakage assay

The permeability of the BBB was analyzed using an FITC-albumin leakage assay, as previously described (15). Animals were injected intravenously with 100 mg/kg FITC-albumin 1.5 h after lipopolysaccharide injection (i.p.). At 2.5 h after the FITC-albumin injection, the mice were anesthetized and the brains were harvested. The left hemi-brains were fixed with paraformaldehyde and cut using a cryostat at a 40-μm thickness for histological analysis. The right hemi-brains were homogenized in five volumes (wt/vol) of cold PBS with a Teflon-glass homogenizer (Thomas Scientific, Swedesboro, NJ, USA). The samples were centrifuged at 10,000 × g for 30 min. The supernatant was collected and the optical densities of the homogenates supernatant were read at 488 nm excitation and 525 nm emission with a fluorescent plate reader (Ascent, Thermo Scientific, Waltham, MA, USA).

### Polymerase chain reaction (PCR)

The toes from all mice were removed according to toe numbering scheme (http://research.fhcrc.org/fero/en/fero-lab-protocols/mouse-toe-identification.html) at the postnatal stage P7 and immediately frozen on dry ice. DNA was extracted using protocols provided by the Dr Yu-Qiang Ding of Tongji University. The extracted DNA was stored at −20°C until required. TPH2 was analyzed using a PCR detection system (Biometra 070–851; Analytik Jena, Jena, Germany). The TPH2 or Cre gene fragments were amplified using previously described primers (11). The 10 μl total reaction mixture contained 1 μl genomic DNA, 1 μl of each primer, 3.5 μl 2xTaq PCR MasterMix (Tiangen Biotech, Co., Ltd., Beijing, China) and 3.5 μl ddH_2_O. The reaction mixture was initially denatured at 94°C for 2 min, followed by 30 cycles at 94°C for 30 sec, 58–60°C for 30 sec and 72°C for 45 sec. The PCR was completed by a final extension cycle at 72°C for 5 min. Successful amplification of the fragments was confirmed by detection of a 213 or 384 bp band for Tph2, or 400 bp strand for Cre on a 1.5% agarose gel.

### Statistical analysis

All data are expressed as the mean ± standard deviation. Two-group comparisons were performed by the Student’s t-test. P<0.05 was considered to indicate a statistically significant difference.

## Results and Discussion

Disturbances in the BBB are becoming a common denominator in depressive disorders (3,4). A dysfunctional BBB can lead to the leakage of various neurotoxic substances into the brain, resulting in neuronal damage (14) and brain dysfunction. In the present study, it was demonstrated using EB staining that BBB impairment does not occur in TPH2-knockout mice.

**Figure 1:**
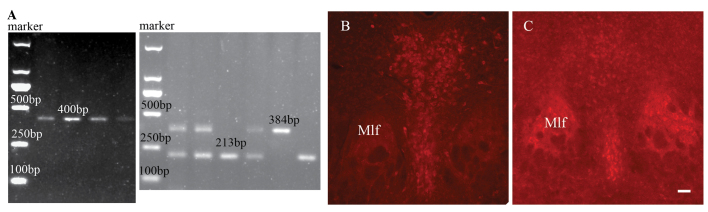
Polymerase chain reaction and immunocytochemistry results. (A) Expression of the Cre (left) and TPH2 (right) marker genes. (B) Immunofluorescence in 5-HT-positive neurons in the wild-type group (magnification, ×20). (C) 5-HT-positive neurons were lost in the TPH2-knockout group (magnification, ×20). Scale bar=20 μm. Mlf, medial longitudinal fasciculus; 5-HT, 5-hydroxytryptamine; TPH2, tryptophan hydroxylase 2.

In order to ensure that the TPH2 gene was knocked out in the experiment, PCR and immunocytochemistry methods were performed, as illustrated in Fig. 1. Mice were genotyped by PCR with primers against Cre (forward, TCG ATG CAA CGA GTG ATGAG; reverse, TCC ATG AGT GAA CGA ACC TG), resulting in a 400-bp product, as well as against TPH2-flox (forward, CAG GTA GAG AGC CAA TCA AAG AGTG; reverse, CTG GGC TGG CCG ATA GTA ACAC), resulting in 213-bp wild-type and 384-bp heterozygote products. All mice carrying the Cre gene were found to be viable, without any evident abnormalities. Fig. 1A shows the DNA detection results of the wild-type and homozygous TPH2-knockout mice (Cre, 400 bp; wild-type, 213 bp; and heterozygous TPH2-knockout, 384 bp). TPH2-positive neurons were observed in the wild-type mice (Fig. 1B), but not in the knockout mice (Fig. 1C). Thus, the results showed that all the knockout mice used in the study exhibited TPH2 gene absence.

**Figure 2:**
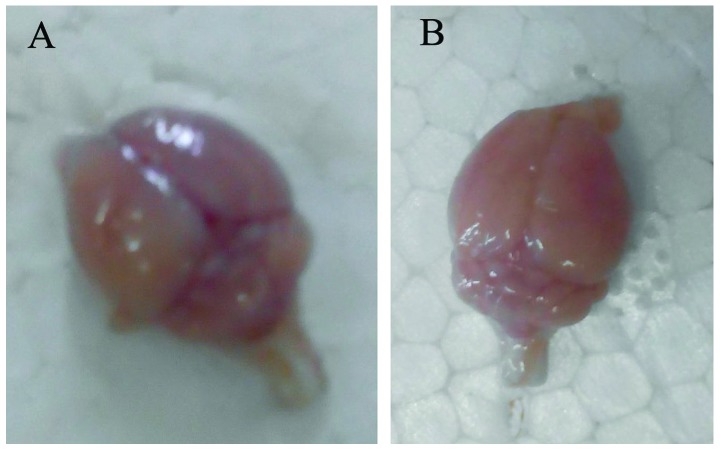
Mouse brains 12 h after Evans Blue intraperitoneal injection. (A) Wild-type group. (B) Tryptophan hydroxylase 2-knockout group.

**Figure 3:**
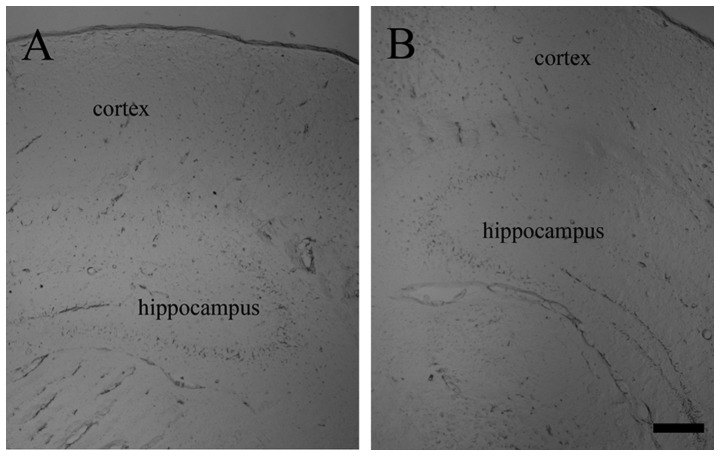
Mouse brain coronal sections 12 h after Evans Blue intraperitoneal injection (magnification, ×20). (A) Wild-type group. (B) Tryptophan hydroxylase 2-knockout group. Scale bar=40 μm.

As shown in Fig. 2A and B, no EB stain could be observed in the whole brain in the TPH2-knockout or wild-type groups after 12 h EB i.p. injection. Similarly, no differences were observed in the tissue sections between the two groups (Fig. 3). Notably, when the mouse abdominal spaces were open, it was observed that the lung, heart, kidney and liver were all stained blue by EB. Furthermore, in order to verify the effect of the TPH2 gene on the BBB integrity, the permeability of the BBB was examined using the FITC-albumin leakage assay. FITC-labeled albumin was injected from the tail vein, and leakage of dye into the brain parenchyma was measured as an index of BBB permeability. FITC-albumin was restricted to the inside of the brain blood vessels and no significant signals were detected in the brain parenchyma in the two genotypes following PBS injection (data not shown). Quantification of the FITC-albumin leakage revealed that the severity of the BBB breakdown was not significantly different in the TPH2-knockout and wild-type mice. Therefore, it was speculated that the knockout of the TPH2 gene had no effect on the BBB. By contrast, BBB permeability has been shown to be influenced by the elevation of circulating 5-HT levels (16), neutralization of endogenous 5-HT activity and/or the blocking of its receptors (6,17). Therefore, the data on the effect of serotonin on BBB permeability are contradictory. This may be due to differences in the species used, the dose regimen applied or the animal model.

The BBB is known to change in major depressive disorder (MDD), which is a severe psychiatric syndrome with a high prevalence and socioeconomic impact (18). MDD (lifetime prevalence 13–16%) is also a complex combination of disturbances in cognition, behaviour and physical functioning. MDD is the third leading cause of global disease and a leading cause of disability worldwide as depression is clinically and aetiologically heterogeneous (19). However, the underlying pathophysiology of MDD has yet to be fully elucidated. Despite this, it has been indicated that a disturbance in central 5-HT activity is a key factor (18). In the present study, when TPH2 was knocked out and the 5-HT neurons were lost, abnormal behavior was observed, but no difference was identified in the EB staining. Thus, we hypothesized that BBB permeability in 5-HT-related MDD is influenced by a number of factors (4).

In conclusion, the 5-HT system offers numerous possibilities to develop novel treatments for MDD. Understanding the association between TPH2 (a 5-HT synthesis rate-limiting enzyme in the central nervous system) and BBB permeability may be beneficial for the identification of novel therapeutic and preventative approaches in MDD.
